# Ginsenoside Rg1 Exerts Anti-inflammatory Effects via G Protein-Coupled Estrogen Receptor in Lipopolysaccharide-Induced Microglia Activation

**DOI:** 10.3389/fnins.2019.01168

**Published:** 2019-11-07

**Authors:** Xian-Qi Gao, Zhong-Rui Du, Liang-Jie Yuan, Wen-Di Zhang, Lei Chen, Ji-Jun Teng, Man-Sau Wong, Jun-Xia Xie, Wen-Fang Chen

**Affiliations:** ^1^Department of Physiology, Shandong Key Laboratory of Pathogenesis and Prevention of Neurological Disorders and State Key Disciplines: Physiology, School of Basic Medicine, Qingdao University, Qingdao, China; ^2^School of Basic Medicine, Shandong First Medical University and Shandong Academy of Medical Sciences, Tai’an, China; ^3^Department of Neurology, Affiliated Hospital of Qingdao University, Qingdao, China; ^4^Department of Applied Biology and Chemical Technology, The Hong Kong Polytechnic University, Hung Hom, China

**Keywords:** ginsenoside Rg1, G protein-coupled estrogen receptor, lipopolysaccharide, neuroinflammation, microglial cells

## Abstract

Neuroinflammation plays a pivotal role in the pathogenesis of Parkinson’s disease. Ginsenoside Rg1, the most active ingredient of ginseng, has been reported to exert neuroprotective effects via estrogen and glucocorticoid receptors. The present study evaluated the involvement of the G protein-coupled estrogen receptor (GPER) in the anti-inflammatory effects of ginsenoside Rg1 against lipopolysaccharide (LPS)-induced microglia activation in the BV2 microglial cell line and ventral mesencephalic primary microglial culture. The pharmacological blockade and lentivirus-mediated small interfering RNA (siRNA) knockdown of GPER were used to study the underlying mechanism. Rg1 attenuated LPS-induced upregulation of tumor necrosis factor-α (TNF-α), interleukin-1β (IL-1β), inducible nitric oxide synthase (iNOS), and cyclooxygenase-2 (COX-2) mRNA and protein levels. The GPER antagonist G15 blocked the inhibitory effects of Rg1 and the GPER-specific agonist G1 on LPS-induced microglia activation. Rg1 mimicked the effects of G1 by inhibiting the LPS-induced activation of nuclear transcription factor-kappa B (NF-κB) and mitogen activated protein kinase signaling pathways, which was also blocked by G15. Moreover, lentivirus-mediated siRNA knockdown of GPER inhibited the anti-inflammatory effects of Rg1. Taken together, our results indicate that GPER is involved in the anti-inflammatory effects of Rg1 against LPS-induced microglia activation. These findings provide a new biological target of Rg1 for the treatment of neuroinflammatory disorders.

## Introduction

Several studies have suggested that neuroinflammation plays an important role in the etiology of neurodegenerative disorders, including Parkinson’s disease, Alzheimer’s disease, multiple sclerosis, and encephalitides (Nolan et al., 2013; Heppner et al., 2015; Borjini et al., 2016; Schwartz and Deczkowska, 2016). Microglia has been recognized as the main actor in the neuroinflammatory responses in the central nervous system (Wolf et al., 2017). Over-activation of microglia, which is accompanied by multiple inflammatory reactions, is considered deleterious to the surrounding neurons (Hirsch and Hunot, 2009). Inhibition of over-activated microglia might therefore be a beneficial therapy to delay or even reverse the development of neuroinflammatory diseases (Wang et al., 2015; Le et al., 2016).

The ER belongs to the superfamily of nuclear receptors. The genomic effect of estrogen is mediated by the classical nuclear receptors ERα and ERβ (Prossnitz and Arterburn, 2015). The GPER, a 7-transmembrane receptor, was first reported to have an estrogen binding affinity in Revankar et al. (2005). More recently, the GPER was found to be widely distributed in the reproductive, cardiovascular, musculoskeletal, and nervous systems (Prossnitz and Barton, 2011; Filardo and Thomas, 2012). The GPER mediates the non-genomic effect of estrogen and plays an important role in the anti-inflammatory and anti-apoptotic effects of estrogen (Tamaki et al., 2014; Zhao et al., 2016). G1, a GPER selective agonist, can inhibit the production of LPS-induced cytokines in the animal model of multiple sclerosis and Parkinson’s disease (Blasko et al., 2009; Mendes-Oliveira et al., 2017). This arouses the possibility that the anti-inflammatory function of GPER could be exploited in the treatment of neuroinflammatory diseases.

Ginsenoside Rg1 is the major pharmacological active ingredient of traditional Chinese medicine ginseng with a four trans-ring rigid steroid skeleton structure (Leung et al., 2007; Lu et al., 2015). Our previous studies have demonstrated that Rg1 is a novel phytoestrogen (Chan et al., 2002). Rg1 can increase estrogen responsive element luciferase activity and induce estrogen-dependent pS2 gene expression. A receptor binding assay found that Rg1 did not bind to the classical nuclear receptors ERα and ERβ (Chan et al., 2002; Gao et al., 2009). The estrogenic activity of Rg1 in the absence of direct binding with ERα and ERβ indicates that Rg1 may activate ER in a ligand-independent manner (Shi et al., 2012).

Accumulating research has focused on the functional cross-talk between estrogen signaling and the growth factor system. Activation of GPER can induce the activation of the insulin-like growth factor-I receptor (IGF-IR) and EGFR (Lappano et al., 2013). Our previous data clearly show that Rg1 can protect against 6-hydroxydopamine (6-OHDA) as well as Aβ25-35-induced toxicity via IGF-IR and ER pathways (Xu et al., 2009; Chen et al., 2013). Zong et al. (2012) reported that Rg1 can protect against LPS-induced inflammation in murine BV2 microglial cells via the phospholipase C LPS-induced pathway. Moreover, Rg1 can also inhibit LPS-induced microglial activation in the cerebral cortex and hippocampus in mice (Hu et al., 2011). But the detailed mechanism of the anti-inflammatory effect of Rg1 is unclear. In the present study, we hypothesized that Rg1 exerts its anti-inflammatory effects via the GPER. Thus, we examined the anti-inflammatory effects of Rg1 in an LPS-induced microglial cell model. Pharmacological blockade and small interfering RNA (siRNA) knockdown of the GPER were used to elucidate the role of GPER activation on the anti-inflammatory effects of Rg1.

## Materials and Methods

### Experimental Materials

Ginsenoside Rg1 (purity > 99%) was supplied by Jilin University (Changchun, China). LPS (from *Escherichia coli*) was obtained from Sigma (St. Louis, MO, United States). G1 (3577) and G15 (3678) were purchased from Tocris Bioscience (Bristol, United Kingdom). Rabbit polyclonal antibodies against GPER, iNOS (ab3523), COX-2 (ab15191), and β-actin (ab8227) were purchased from Abcam (Cambridge, United Kingdom). Primary antibodies against phospho-inhibitor of NF-κB (phospho-IκB, #2859), IκB (#4812), phospho-extracellular signal-regulated kinase 1/2 (phospho-ERK1/2, #9101), ERK1/2 (#9102), phospho-p38 (#4511), p38 (#9212), phospho-c-Jun-N-terminal kinase (phospho-JNK, #9251), JNK (#9252), and secondary antibodies of rabbit anti-goat IgG-horseradish peroxidase (HRP, sc-2054) were provided by Santa Cruz Biotechnology, Inc. (Santa Cruz, CA, United States). Alexa Fluor 555-conjugated anti-rabbit IgG antibodies and Alexa Fluor 488-conjugated anti-mouse IgG antibodies were supplied by Thermo Scientific (United States).

### BV2 Microglial Cell Culture

BV2 microglia is a murine immortalized microglia cell line that had been proven as a valid substitute for primary microglia (Henn et al., 2009). BV2 microglia cells were purchased from the Cell Resource Center of IBMS, CAMS/PUMC (3111C0001CCC000063, Beijing, China, RRID: CVCL_0182) and cultured in Dulbecco’s modified Eagle’s medium (DMEM) with high glucose, supplemented with 10% heat-inactivated fetal bovine serum, 100 U mL^–1^ penicillin, and 100 μg mL^–1^ streptomycin (Invitrogen, Carlsbad, CA, United States) in a humidified incubator at 37°C in a mixture of 95% air and 5% CO_2_. BV2 cells were utilized for experiments at 70–80% confluence.

### Ventral Mesencephalic Primary Microglia Cell Culture

Animals were obtained from the Experimental Animal Center of Shandong. Animal handling was carried out according to the ethical regulations and guidelines (Guide for the Care and Use of Laboratory Animals, NIH Publications No. 8023, revised 1978) and the European Communities Council Directive 86/609/EEC.

Primary rat ventral mesencephalic microglia culture was performed as previously described (Panicker et al., 2015). Briefly, ventral mesencephalic tissues were dissected from 1-day-old Sprague Dawley rats and dissociated using a mild mechanical trituration. Then, dissociated cells were seeded into the cell culture bottles (125 cm^2^) precoated with poly-D-lysine (20 mg ml^–1^). The culture medium was DMEM-H supplemented with 10% fetal bovine serum and 1% penicillin–streptomycin. Then, microglia were purified from the mixed cell culture; the enriched microglia were > 95% pure, as determined by the microglial marker CD11b antibody. All animal procedures performed in this study were reviewed and approved by the Animal Ethic Committee of Qingdao University, and the protocols were approved by the Institutional Animal Care and Use Committee (IACUC) of Qingdao University. All efforts were made to minimize the number of animals used and their suffering.

### Immunocytofluorescence

Immunocytofluorescence was used to detect the expression of GPER in BV2 microglial cells and primary microglia. Cells were plated onto poly-L-lysine-coated glass slides and fixed with 4% paraformaldehyde in phosphate-buffered saline. Cells were then permeabilized with 0.1% Triton X-100 and incubated with anti-GPER polyclonal antibody (1:250) and anti-CD11b polyclonal antibody (1:250). Alexa Fluor 555-conjugated anti-rabbit IgG antibodies and Alexa Fluor 488-conjugated anti-mouse IgG antibodies were used as secondary antibodies. Hochest 33258 was used to stain the cell nuclei. Stained cells were observed and photographed under a Zeiss AX10 Observer A1 microscope.

### Real-Time PCR

Total RNA was isolated from the microglial cells using TRIzol reagent (Invitrogen, Carlsbad, CA, United States) according to the manufacturer’s instructions. A total of 2 μg of total RNA was reverse-transcribed using the Transcriptor First Strand cDNA Synthesis kit (Roche, Manheim, Germany) to produce the cDNA. The primers were designed and synthesized by Takara Biotechnology, Co., Ltd. The mouse primer sequences were as follows: GPER forward primer 5′-CCCTTAAGCTGCTGGAATTGTG-3′ and reverse primer 5′-AATCGTCCTGGGAGCCTGTTAG-3′; iNOS forward primer 5′-CAAGCTGAACTTGAGCGAGGA-3′ and reverse primer 5′-TTTACTCAGTGCCAGAAGCTGGA-3′; COX-2 forward primer 5′-CTGGAACATGGACTCACTCAGTTTG-3′ and reverse primer 5′-AGGCCTTTGCCACTGCTTGTA-3′; TNF-α forward primer 5′-TATGGCCCAGACCCTCACA-3′ and reverse primer 5′-GGAGTAGACAAGGTACAACCCATC-3′; interleukin-1β (IL-1β) forward primer 5′-TCCAGGATGAGGACATGAGCAC-3′ and reverse primer 5′-GAACGTCACACACCAGCAGGTTA-3′; and GAPDH forward primer 5′-TGTGTCCGTCGTGGATCTGA-3′ and reverse primer 5′-TTGCTGTTGAAGTCGCAGGAG-3′. The rat primer sequences were as follows: iNOS forward primer 5′-GGGACCTGGCTTCCTTGTT-3′ and reverse primer 5′-CCAGCAGTAGTTGTTCCTCTTCC-3′; COX-2 forward primer 5′-ACACGGACTTGCTCACTTTGTT-3′ and reverse primer 5′-TGGTATTTCATCTCTCTGCTCTGG-3′; TNF-α forward primer 5′-TGGGCTCCCTCTCATCAGTT-3′ and reverse primer 5′-TGCTCCTCTGCTTGGTGGT-3′; IL-1β forward primer 5′-CCCTGAACTCAACTGTGAAATAGCA-3′ and reverse primer 5′-CCCAAGTCAAGGGCTTGGAA-3′; and GAPDH forward primer 5′-GGCACAGTCAAGGCTGAGAATG-3′ and reverse primer 5′-ATGGTGGTGAAGACGCCAGTA-3′. Real-time PCR was performed using the QuantiFast SYBR Green RT-PCR Kit (QIAGEN, Germantown, MD, United States). An initial denaturation step at 95°C for 5 min was followed by 40 cycles of denaturation at 95°C for 10 s and annealing at 60°C for 30 s. A melting curve was performed to analyze the PCR products. These steps were carried out by an Eppendorf Mastercycler PCR machine. A comparative threshold cycle (CT) method was used, and the PCR data were calculated using the equation 2^–ΔΔCT^. Each sample was processed in duplicate.

### Enzyme-Linked Immunosorbent Assays

The protein levels of TNF-α and IL-1β were detected by enzyme-linked immunosorbent assay (ELISA kit, R&D Systems) according to the manufacturer’s instructions. BV2 microglial cell culture supernatant was collected and spun down at 1,000 g to eliminate cell debris. The optical density of each well was determined at 450 nm in a microplate reader (Spectra Max M5). The sample levels were calculated from a standard curve and were corrected for protein concentration.

### Immunoblotting

The microglial cells were lysed in radioimmunoprecipitation assay (RIPA) lysis buffer (Beyotime Institute of Biotechnology, Haimen, China) supplemented with 1% PMSF. After centrifugation (12,000 rpm for 15 min at 4°C), the supernatants were collected, and the protein concentrations in the supernatants were analyzed using a BCA colorimetric protein assay kit (Thermo Scientific, United States). A total of 20 μg protein was separated by 10% SDS-PAGE and transblotted onto PVDF membranes (Immobilin-P, Millipore, Corp., Billerica, MA, United States). The membranes were blocked with 5% skimmed milk powder for 1 h at room temperature and then incubated separately with primary antibodies for ERK1/2 and phospho-ERK1/2, JNK and phospho-JNK, p38 MAPK and phospho-p38 MAPK, IκB and phospho-IκB (1:1000), iNOS (1:3000), COX-2 (1:1000), GPER (1:1000), p65 and p-p65 (1:1000), and β-actin (1:5000) overnight at 4°C. After washing the membranes with TBS and Tween 20, the membranes were incubated with rabbit secondary HRP-linked antibodies (Santa Cruz) for 2 h at room temperature. The antigen–antibody complexes were detected with enhanced chemiluminescence (ECL) reagent (Millipore) and visualized by Imager (UVP Biospectrum 810, United States).

### Construction and Transfection of Lentivirus-Mediated Small Interfering RNA

Lentivirus-mediated siRNA was designed by Shanghai GeneChem, Co., Ltd., China. To generate lentivirus-expressing RNAi specific for the GPER gene (NM_029771), the RNA interference sequence for the mouse GPER (CTATTGGCTTTGTGGGCAA) and the negative control sequence (TTCTCCGAACGTGTCACGT) were cloned into the hU6-MCS-CMV-EGFP to generate the GV115 lentiviral vectors.

To infect BV2 microglial cells, lentivirus particles were added to the culture medium at a multiplicity of infection of 100. The cells were divided into a control (Lv-siCon) group and a lentivirus (Lv-siGPER) group. In the Lv-siCon group, we treated the cells only with lentivirus vector, which did not link with target gene. Cells were then cultured in the incubator at 37°C and in 5% CO_2_. The efficiency of infection was determined by GFP percentage under fluorescence microscopy 72 h after lentivirus infection. siRNA knockdown efficiency was determined by real-time PCR and immunoblotting on GPER mRNA and protein levels, respectively.

### Statistical Analysis

Data are presented as the mean ± SEM. Statistical analysis was carried out using GraphPad Prism Software 5.0, United States. Different groups were analyzed by one-way ANOVA followed by Tukey’s *post hoc* tests. Comparisons between the Lv-siCon group and the Lv-siGPER group were made using unpaired Student’s *t*-tests. A two-way ANOVA followed by *post hoc* Bonferroni/Dunn tests were used to analyze the statistical significance for the transfection experiments. *P* < 0.05 was considered significant.

## Results

### Both BV2 Microglial Cells and Primary Microglia Expressed GPER

**FIGURE 1:**
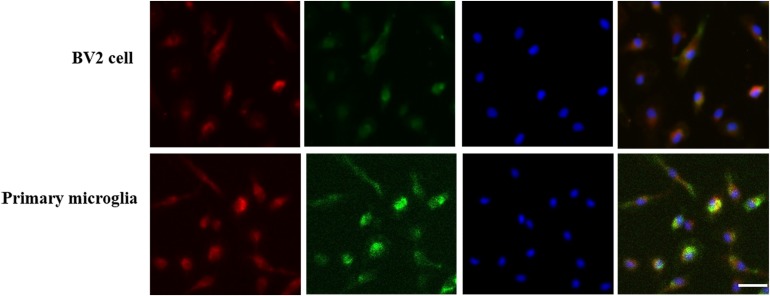
G protein-coupled estrogen receptors co-localized with CD11b in BV2 cell and primary microglia. GPER was highly expressed in BV2 cell and primary microglia. Three-labeling immunofluorescence showed the co-localization of GPERs (red) with CD11b (green) and the cell nuclei (blue) in BV2 cells and primary microglia. Scale bar = 100 μm.

As shown in Figure 1, BV2 cells and primary microglia presented a strong immunoreactivity against the GPER and CD11b, confirming the protein expression of GPER in microglial cells.

### Different Dosages of Rg1 Inhibited LPS-Induced Upregulation of TNF-α, IL-1β, iNOS, and COX-2 in BV2 Cells

**FIGURE 2:**
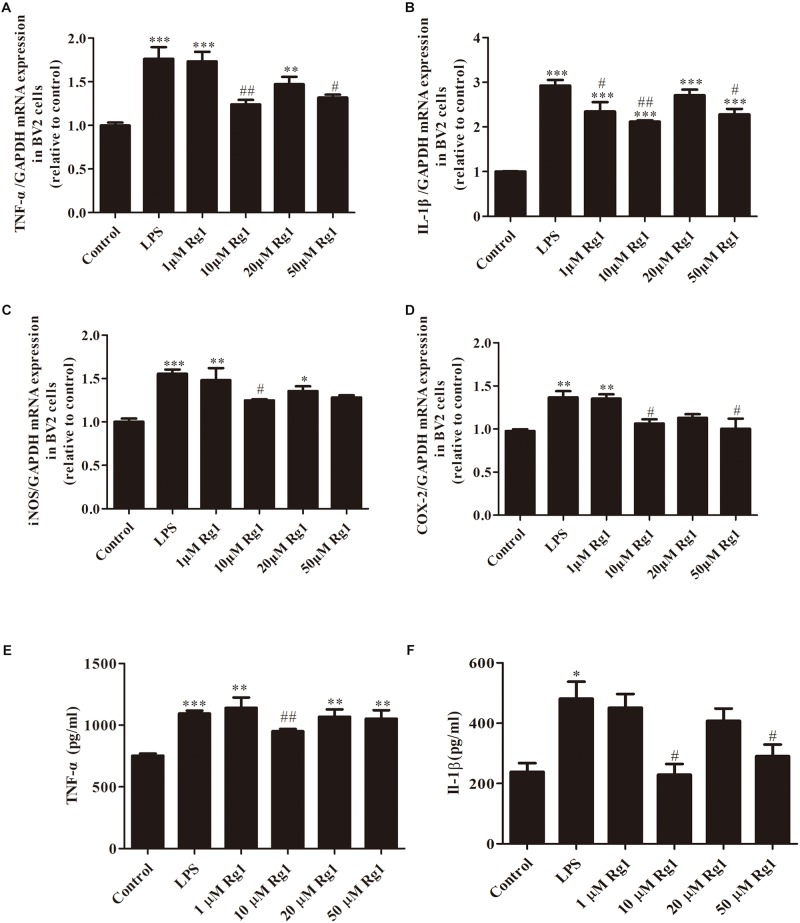
The inhibitory effects of different dosages of Rg1 on LPS-induced production of TNF-α, interleukin-1β (IL-1β), iNOS, and COX-2 in BV2 cells. BV2 cells were treated with the indicated concentration of Rg1 (1, 10, 20, and 50 μM) 1 h before LPS (1 μg ml^–1^) treatment for 6 h. TNF-α **(A)**, IL-1β **(B)**, iNOS **(C)**, and COX-2 **(D)** mRNA levels were measured by real-time PCR; GAPDH was used as an internal control (*n* = 4). TNF-α **(E)** and IL-1β **(F)** protein levels were detected by ELISA (*n* = 3). Results are expressed as the mean ± SEM. ^∗^*P* < 0.05, ^∗∗^*P* < 0.001, and ^∗∗∗^*P* < 0.001 versus the control; ^#^*P* < 0.05 and ^##^*P* < 0.01 versus the LPS group.

Proinflammatory cytokines released from activated microglia exert an important role in neuronal damage (Liu and Bing, 2011; Chung et al., 2012). To demonstrate the anti-inflammatory effects of Rg1, the mRNA expressions of TNF-α, IL-1β, iNOS, and COX-2 were detected by real-time PCR. The mRNA expressions of TNF-α, IL-1β, iNOS, and COX-2 were significantly increased in LPS-treated BV2 microglial cells when compared to the control group. Pretreatment with different dosages of Rg1 (1, 10, 20, and 50 μM) inhibited LPS-induced inflammatory responses (Figures 2A–D). The release of TNF-α and IL-1β was determined by ELISA (Figures 2E,F). 10 μM Rg1 could significantly decrease the release of TNF-α and IL-1β. The most effective dosage of Rg1 is 10 μM. In the following experiment, which was conducted to determine the underlying mechanism of the anti-inflammatory effects of Rg1, 10 μM Rg1 was chosen.

### The GPER Was Involved in the Anti-inflammatory Effect of Rg1 in Microglial Cells

**FIGURE 3:**
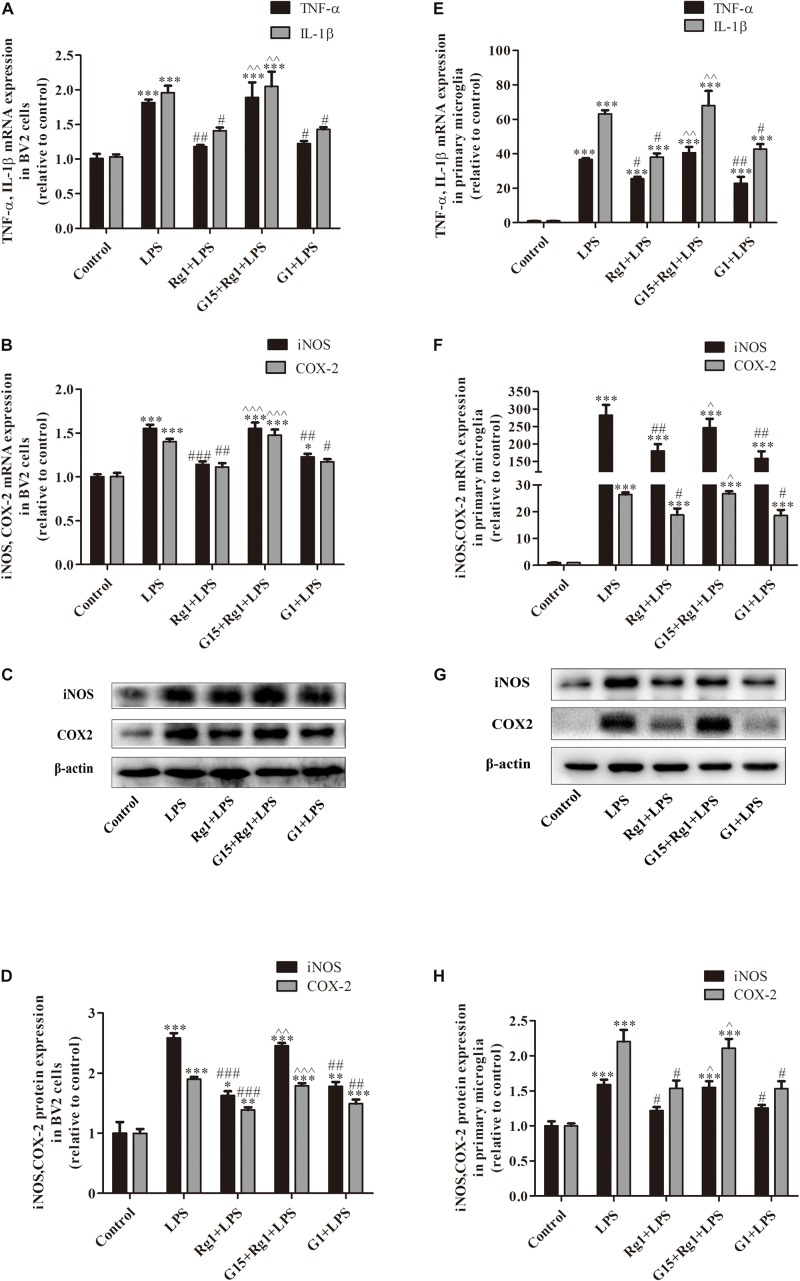
The GPER was involved in the inhibitory effects of Rg1 on mRNA and protein expression of proinflammatory cytokines in BV2 cells and primary microglia. BV2 cells **(A–D)** and primary microglial cells **(E–H)** were pretreated with 1 μM G15 for 1 h, followed by 10 μM Rg1 or 1 μM G1 for 1 h. LPS (BV2 cells: 1 μg ml^–1^; primary microglia: 0.5 μg ml^–1^) was used to stimulate the cells for 6 h to collect the total RNA or for 24 h to detect protein expression. TNF-α, IL-1β, iNOS, and COX-2 mRNA levels were measured using real-time PCR. GAPDH was used as an internal control. Western blot was used to detect iNOS and COX-2 protein levels. β-Actin was used as an internal control. Results are expressed as the mean ± SEM, *n* = 3–5. ^∗^*P* < 0.05, ^∗∗^*P* < 0.01, and ^∗∗∗^*P* < 0.001 versus control; ^#^*P* < 0.05, ^##^*P* < 0.01, and ^###^*P* < 0.001 versus the LPS group; ^∧^*P* < 0.05, ^∧∧^*P* < 0.01, and ^∧∧∧^*P* < 0.001 versus the Rg1 + LPS group.

To determine the possible contribution of the GPER in the anti-inflammatory effects of Rg1, microglial cells were treated with LPS (BV2: 1 μg ml^–1^, primary microglia: 0.5 μg ml^–1^) in the presence or absence of Rg1 (10 μM) or G15 (1 μM). Real-time PCR analysis showed that G15 pretreatment significantly blocked the inhibitory effects of Rg1 on LPS-induced IL-1β, TNF-α, iNOS, and COX-2 mRNA expressions in BV2 cells (Figures 3A,B) and primary microglial cells (Figures 3E,F). We also investigated whether GPER was associated with the anti-inflammatory effects of Rg1 on COX-2 and iNOS protein expressions. Western blot analysis showed that G15 significantly antagonized the inhibitory effects of Rg1 on COX-2 and iNOS protein expressions in BV2 (Figures 3C,D) and primary microglial cells (Figures 3G,H). G1 (1 μM), a specific agonist of GPER, also significantly inhibited the LPS-induced mRNA and protein expression of proinflammatory cytokines in BV2 and primary microglial cells.

### Rg1 Inhibited the LPS-Induced Activation of the MAPK Signaling Pathways via the GPER in Microglial Cells

**FIGURE 4:**
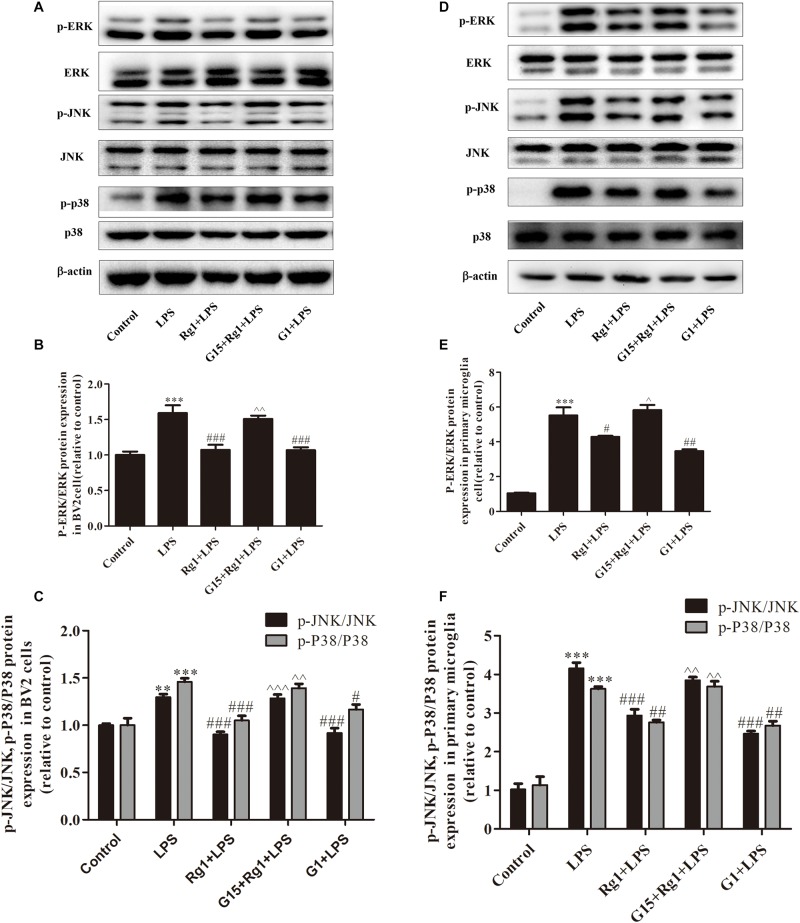
The GPER was involved in the anti-inflammatory effects of Rg1 on the activation of MAPK signaling pathway in BV2 cells and primary microglia. BV2 cells and primary microglia were incubated in DMEM-H without FBS for 4 h, pretreated with 1 μM G15 for 1 h, followed by 10 μM Rg1 or 1 μM G1 for 1 h, and stimulated with LPS (BV2 cells: 1 μg ml^–1^; primary microglia: 0.5 μg m^–1^) for 0.5 h. Cell lysates were prepared and subjected to western blot. The phosphorylation level of ERK, c-Jun-N-terminal kinase (JNK), and p38 were detected by western blot in BV2 cells **(A–C)** and primary microglia **(D–F)**. Equivalent loading of cell lysates was determined by the total ERK, JNK, and P38. Results are expressed as the mean ± SEM, *n* = 3–4. ^∗∗^*P* < 0.01 and ^∗∗∗^*P* < 0.001 versus control; ^#^*P* < 0.05, ^##^*P* < 0.01, and ^###^*P* < 0.001 versus the LPS group; ^∧^*P* < 0.05, ^∧∧^*P* < 0.01, and ^∧∧∧^*P* < 0.001 versus the Rg1 + LPS group.

Mitogen activated protein kinase signal transduction involves p38, ERK, and JNK, which are the most important signaling molecules known to regulate gene expression of proinflammatory cytokines in microglial over-activation. The BV2 (Figures 4A–C) and primary cultured microglial cells (Figures 4D–F) were treated with LPS for 0.5 h in the presence or absence of Rg1. The protein phosphorylation of ERK, JNK, and p38 induced by LPS was significantly inhibited by Rg1. These effects could be blocked by G15. G1 also significantly inhibited the LPS-induced phosphorylation of ERK, JNK, and p38.

### GPER Mediated the Inhibitory Effects of Rg1 on the Activation of Nuclear Transcription Factor-Kappa B Signaling Pathway in Microglia Cells

**FIGURE 5:**
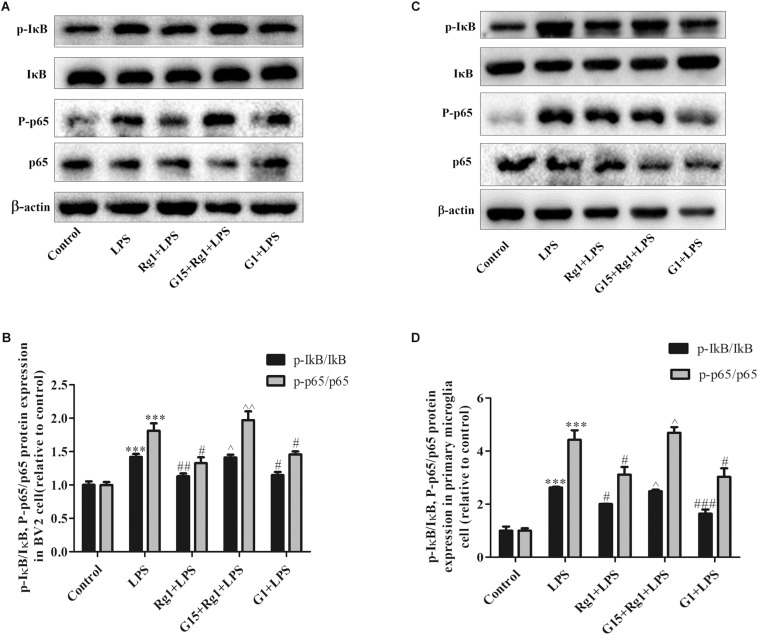
G15 blocked the inhibitory effect of Rg1 on LPS-induced activation of nuclear transcription factor-kappa B (NF-κB) signaling pathway. BV2 cells and primary microglia were incubated in Dulbecco’s modified Eagle’s medium (DMEM)-H without FBS for 4 h, pretreated with 1 μM G15 for 1 h, followed by 10 μM Rg1 or 1 μM G1 for 1 h, and stimulated with LPS (BV2 cells: 1 μg ml^–1^; primary microglia: 0.5 μg ml^–1^) for 0.5 h. The phosphorylation levels of inhibitor of NF-κB (IκB) and p65 were detected by western blot in BV2 cells **(A,B)** and primary microglia **(C,D)**. Results are expressed as the mean ± SEM, *n* = 3. ^∗∗∗^*P* < 0.001 versus control; ^#^*P* < 0.05, ^##^*P* < 0.01, and ^###^*P* < 0.001 versus the LPS group; ^∧^*P* < 0.05 and ^∧∧^*P* < 0.01 versus the Rg1 + LPS group.

Nuclear transcription factor-kappa B (NF-κB) pathway is also involved in the LPS-induced microglia activation. NF-κB P65 protein and its inhibitor IκB were detected by western blot. As shown in Figures 5A–D, Rg1 pretreatment significantly inhibited the LPS-induced phosphorylation of IκB and p65 in BV2 and primary cultured microglial cells. Co-treatment with G15 could inhibit the protective effects of Rg1.

### GPER Silencing Attenuated the Inhibitory Effects of Rg1 on LPS-Induced TNF-α and IL-1β Gene Expression in BV2 Cells

**FIGURE 6:**
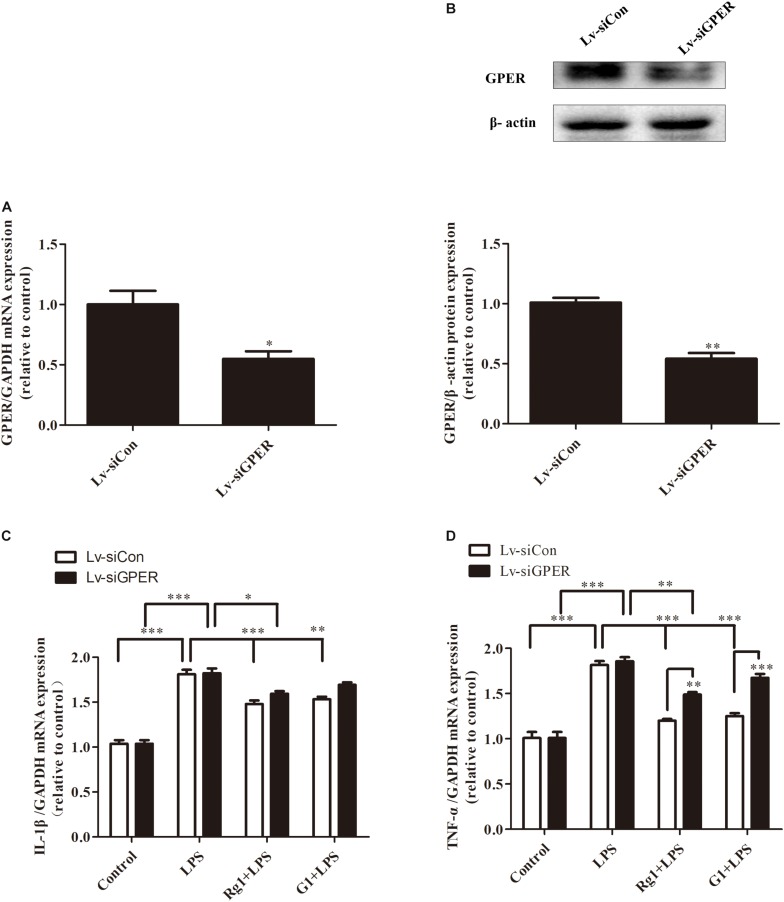
G protein-coupled estrogen receptor gene silencing attenuated the anti-inflammatory effects of Rg1 on TNF-α and IL-1β mRNA expression in BV2 cells. BV2 cells were infected with Lv-siCon and Lv-siGPER for 72 h separately. The mRNA and protein expression of GPER in the Lv-siCon and Lv-siGPER groups were detected by real-time PCR **(A)** and western blot **(B)**. Glyceraldehyde-3-phosphate dehydrogenase and β-actin were used as internal control. Results are expressed as the mean ± SEM, *n* = 3. The analyses were carried out using unpaired Student’s *t*-tests. ^∗^*P* < 0.05 and ^∗∗^*P* < 0.01 versus the control. BV2 cells were infected with Lv-siCon and Lv-siGPER for 72 h separately, followed by 10 μM Rg1 or 1 μM G1 for 1 h; 1 μg ml^–1^ LPS was used to stimulate the cells for 6 h, and the total RNA was collected. IL-1β **(C)** and TNF-α **(D)** mRNA levels were measured using quantitative real-time PCR. GAPDH was used as an internal control. Results are expressed as the mean ± SEM, *n* = 3–5. ^∗^*P* < 0.05, ^∗∗^*P* < 0.01, and ^∗∗∗^*P* < 0.001 by a two-way ANOVA followed by *post hoc* Bonferroni/Dunn test.

To further demonstrate the involvement of the GPER in the inhibitory effects of Rg1 on LPS-induced mRNA expressions of TNF-α and IL-1β, lentivirus-mediated siRNA was used to knock down the expression of GPER in BV2 microglial cells. Compared with the Lv-siCon group, Lv-siGPER treatment induced a 52.8 and 51.1% reduction in mRNA and protein levels of GPER, respectively (Figures 6A,B). GPER silencing significantly blocked the inhibitory effect of Rg1 on LPS-induced IL-1β and TNF-α mRNA expression (Figures 6C,D), further indicating the involvement of GPER in the anti-inflammatory effect of Rg1. GPER silencing also attenuated the inhibitory effect of G1 on the LPS-induced inflammatory response.

### GPER Silencing Attenuated the Inhibitory Effects of Rg1 on LPS-Induced mRNA and Protein Expression of iNOS and COX-2 in BV2 Cells

**FIGURE 7:**
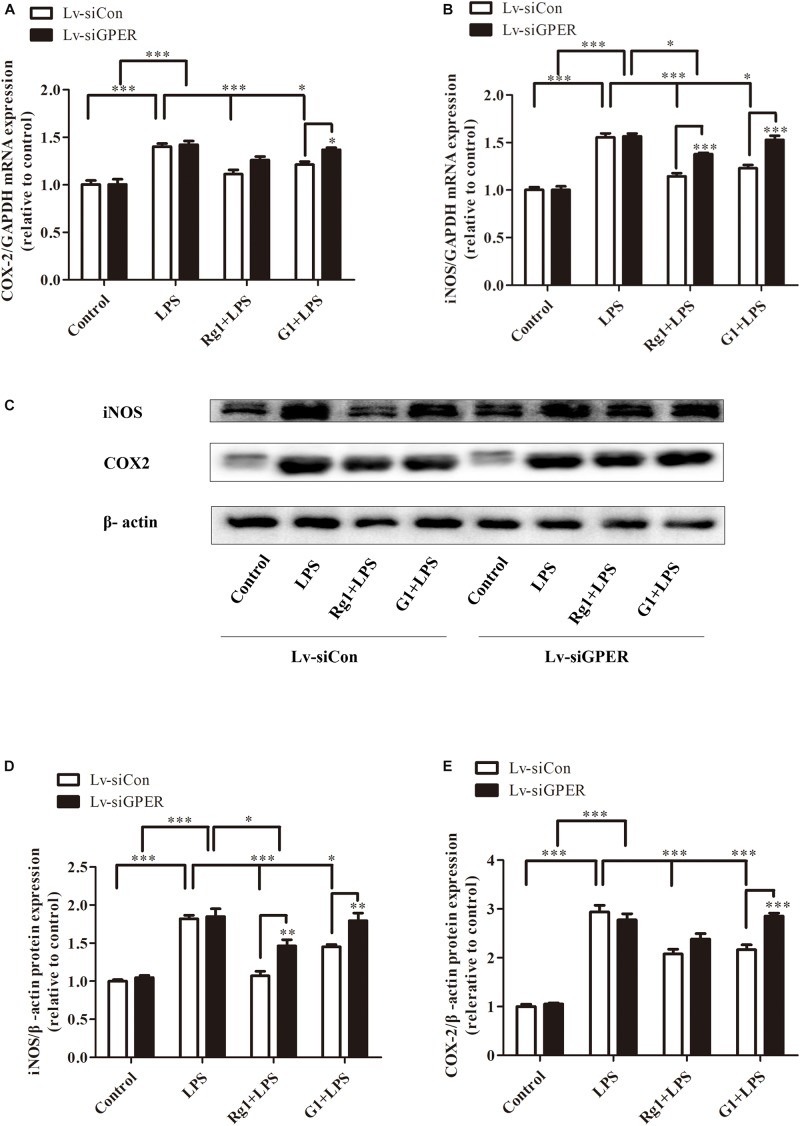
G protein-coupled estrogen receptor gene silencing attenuated the anti-inflammatory effects of Rg1 on iNOS and COX-2 mRNA and protein expression in BV2 cells. BV2 cells were infected with Lv-siCon and LV-siGPER for 72 h separately, followed by 1 μg ml^–1^ LPS for 24 h. Total RNA and cell lysates were prepared to detect the iNOS and COX-2 mRNA and protein levels. COX-2 **(A)** and iNOS **(B)** mRNA levels were measured using quantitative real-time PCR. GAPDH was used as an internal control. Western blot **(C)** was used to detect protein expression of iNOS **(D)** and COX-2 **(E)**. β-Actin was used as an internal control. Results are expressed as the mean ± SEM, *n* = 3–5. ^∗^*P* < 0.05, ^∗∗^*P* < 0.01, and ^∗∗∗^*P* < 0.001 by a two-way ANOVA followed by *post hoc* Bonferroni/Dunn test.

Both real-time PCR and western blot analyses showed that GPER silencing significantly antagonized the inhibitory effects of Rg1 on LPS-induced mRNA (Figures 7A,B) and the protein expression of iNOS and COX-2 (Figures 7C–E). The anti-inflammatory effects of G1 were also blocked by GPER silencing (Figures 7A–E).

## Discussion

**FIGURE 8:**
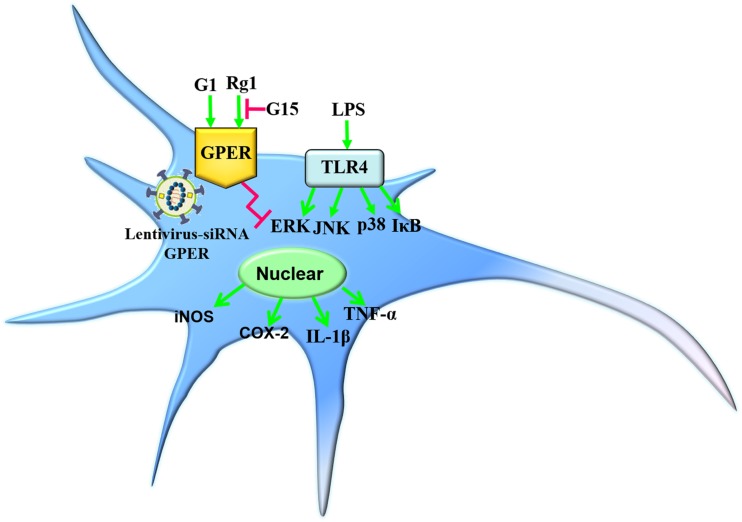
Simplified models depicting the anti-inflammatory effects of Rg1 or G1 against LPS-induced microglia activation. Rg1 and G1 exerted inhibitory effects against the LPS-induced neuroinflammatory response. The pharmacological blockade and lentivirus-mediated siRNA knockdown of GPER significantly inhibited the protective effects of Rg1 or G1.

In the present study, we demonstrated that the anti-inflammatory effects of Rg1 against LPS-induced inflammatory response could be blocked by the GPER antagonist G15. Both Rg1 and G1 treatment significantly inhibited the LPS-induced activation of the NF-κB and MAPK signaling pathways in microglial cells, which were also blocked by G15. Further study confirmed that the pharmacological blockade and lentivirus-mediated siRNA knockdown of GPER significantly inhibited the protective effects of Rg1 and G1 against LPS-induced production of proinflammatory cytokines in microglial cells (Figure 8). The findings reported here indicate that the GPER plays an important role in mediating the anti-inflammatory effects of Rg1.

Lipopolysaccharide was used to induce microglial activation in the current study. As the major component of the cell wall of gram-negative bacteria, LPS can act on the toll-like receptor 4 (TLR4) on the cell surface of microglia to induce a series of activation of the inflammatory signaling pathway and the synthesis and release of inflammatory mediators that activate the downstream signaling (Liu and Bing, 2011; Woller et al., 2016). At the same time, LPS has no direct effect on neurons due to their lack of TLR4, which makes LPS suitable to study the inflammation-mediated neuronal loss (Erny et al., 2015). As expected, LPS treatment induced microglia over-activation by increasing the gene and protein expressions of TNF-α, IL-1β, COX-2, and iNOS. The reactivity of the primary culture microglia to LPS treatment was stronger than the BV2 cell line, even though the LPS dosage in the primary microglia was only half of that in the BV2 cell line. These results are consistent with Henn’s report. The primary microglia may be in a more activated state compared to the BV2 cells. Therefore, primary microglia cells are more sensitive than BV2 cells in response to LPS stimulation (Henn et al., 2009).

The GPER is a newly discovered membrane ER that is widely expressed in the nervous system. Combined with classical ERs, namely, ERα and ERβ, the discovery of GPER has allowed us to expand our knowledge about ERs and to see that the picture is more complex than previously thought in terms of the biological actions of estrogen (Filardo and Thomas, 2012; Prossnitz and Barton, 2014). In the rapid ER signaling pathway, the activation of GPER leads to the activation of adenylate cyclase, phospholipase C, and growth factor receptor, which is followed by a rapid phosphorylation of MAPKs ERK1/2 as well as nuclear translocation of NF-κB (Luo et al., 2012; Bean et al., 2014; Zhu et al., 2017). Studies have shown that the anti-inflammatory effect of estrogen is strongly dependent on the GPER, and the GPER-specific agonist G1 has the same significant therapeutic effects as estrogen in multiple sclerosis (Blasko et al., 2009). In GPER-knockout mice, the protective effects of estrogen and G1 were significantly reduced when compared to GPER-WT mice in experimental autoimmune encephalomyelitis (Wang et al., 2009). Although data have indicated that estrogen has beneficial effects in Parkinson’s disease and the other age-related diseases, many women turn to phytoestrogens as an alternative to hormone replacement therapy (HRT) because of its undesirable side effects (Rossouw et al., 2002). Several lines of evidence have shown that Rg1 is a novel phytoestrogen that exhibits neuroprotective effects both *in vivo* and *in vitro* via the ER and glucocorticoid receptor (Gao et al., 2009; Xu et al., 2009; Wu et al., 2012; Sun et al., 2016). Due to the wide distribution of GPERs in the central nervous system, as well as the multi-target characteristics of Rg1, we focused on the membrane ER GPER. We hypothesized that GPER contributes to the anti-inflammatory actions of ginsenoside Rg1 in microglia in the nigrostriatal system. In agreement with the anti-inflammatory effects of Rg1 reported by Zong et al. (2012), we confirmed the protective effects of Rg1 against LPS-induced inflammatory response. Rg1 treatment could completely block LPS-induced microglia activation in BV2 cells. But in primary culture microglia, Rg1 only partly inhibited the inflammatory response due to the more activated state. The anti-inflammatory effects of Rg1 were blocked by the GPER antagonist G15. Additionally, G1 also inhibited LPS-induced inflammation.

It is well-documented that MAPK and NF-κB signaling pathways are involved in LPS-induced microglia activation. MAPKs, including ERK, JNK, and p38, are crucial in the synthesis and release of inflammatory mediators by activated microglia (Xie et al., 2004; Park et al., 2005). The JNK pathway has been reported to upregulate the protein expression of iNOS in LPS-induced BV2 microglial cells (Svensson et al., 2010). NF-κB is an important transcription factor that induces the expressions of various proinflammatory cytokines. The phosphorylation of IκB can induce the release of NF-κB, which binds to the promoter region of iNOS and the inflammatory cytokine TNF-α in rat primary microglial cells (Fiebich et al., 2002). The present results clearly showed that Rg1 and G1 also suppressed LPS-induced phosphorylation of ERK1/2, JNK, and p38 MAPKs and IκB. Pharmacological blockade of the GPER attenuated the anti-inflammatory effects of Rg1. Therefore, it seems like that GPERs play an important role in mediating the anti-inflammatory effects of Rg1.

To verify the involvement of the GPER in the anti-inflammatory actions of Rg1, lentivirus-mediated siRNA was used to knock down the expression of GPERs in BV2 microglial cells. The efficiency was a 52.8 and 51.1% reduction in mRNA and protein levels, respectively. When compared with the control lentivirus group, the inhibitory effects of Rg1 in the GPER silencing group were significantly decreased, and the anti-inflammatory effects of G1 were completely abolished, which further confirmed the role of GPER in the anti-inflammatory properties of Rg1 and G1 in microglial cells.

## Conclusion

Taken together, the present results indicate that the GPER is involved in the anti-inflammatory effects of ginsenoside Rg1 against LPS-induced microglia activation. These results position GPER as an important target in regulating inflammation and a potential target to protect against inflammatory processes in microglia. These beneficial effects of Rg1 may represent a new strategy for the treatment of neuroinflammatory diseases.

## Data Availability Statement

The raw data supporting the conclusions of this manuscript will be made available by the authors, without undue reservation, to any qualified researcher.

## Ethics Statement

Animal handling was carried out according to ethical regulations and guidelines (Guide for the Care and Use of Laboratory Animals, NIH Publications No. 8023, revised 1978) and the European Communities Council Directive 86/609/EEC. All animal procedures performed in this study were reviewed and approved by the Animal Ethic Committee in Qingdao University and protocols were approved by the Institutional Animal Care and Use Committee (IACUC) in Qingdao University.

## Author Contributions

X-QG, Z-RD, and W-DZ performed the experiments. LC, J-JT, M-SW, J-XX, and W-FC designed the study. X-QG, L-JY, and W-FC drafted the manuscript. All authors read and approved the final manuscript.

## Conflict of Interest

The authors declare that the research was conducted in the absence of any commercial or financial relationships that could be construed as a potential conflict of interest.

## Abbreviations

6-OHDA: 6-hydroxydopamine
COX-2: cyclooxygenase-2
EGFR: epidermal growth factor receptor
ER: estrogen receptor
ERK: extracellular signal-regulated kinase
GAPDH: glyceraldehyde-3-phosphate dehydrogenase
GPER: G protein-coupled estrogen receptor
IGF-IR: insulin-like growth factor-I receptor
IL-1β: interleukin-1β
iNOS: inducible nitric oxide synthase
IκB: inhibitor of NF-κB
JNK: c-Jun-N-terminal kinase
LPS: lipopolysaccharide
MAPK: mitogen activated protein kinase
NF-κB: nuclear factor κB
PI3-K: phosphatidylinositol 3-kinase
TLR4: toll-like receptor 4
TNF-α: tumor necrosis factor-α.
